# The protective effect of proanthocyanidins on the psoriasis-like cell models via PI3K/AKT and HO-1

**DOI:** 10.1080/13510002.2022.2123841

**Published:** 2022-09-30

**Authors:** Yangmeng Zhao, Yuxin Xie, Xiaolong Li, Jing Song, Menglu Guo, Dehai Xian, Jianqiao Zhong

**Affiliations:** a Department of Dermatology, the Affiliated Hospital of Southwest Medical University, Luzhou, People’s Republic of China; b Department of Anatomy, Southwest Medical University, Luzhou, People’s Republic of China

**Keywords:** Psoriasis, proanthocyanidins, inflammation, oxidative stress (OS), PI3K, AKT, HO-1, cell model

## Abstract

**Background:**

Inflammation and oxidative stress (OS) are important contributors to psoriasis pathogenesis. Proanthocyanidins (PCs) have anti-inflammatory and anti-oxidative activities. Previously, we discovered that PCs alleviated psoriasis-like mice symptoms, likely via mitigating inflammation and OS damage.

**Objective:**

To elucidate the protective mechanism underlying PCs against the damage of TNF-ɑ-induced psoriasis-like cell models.

**Methods:**

Psoriasis-like cell models were established with 7.5 ng/mL TNF-ɑ and then subjected to different-concentrations PCs treatment. Finally, inflammatory and oxidative parameters were determined. Besides, LY294002 (PI3K inhibitor) and ZnPP (HO-1 inhibitor) were employed to investigate the roles of PI3K/AKT and HO-1 in PCs against psoriasis-like cell models.

**Results:**

After TNF-α treatment, cells organized tightly and proliferated greatly (P<0.01); HO-1 expression dropped obviously, along with the increased OS/inflammatory indicators and the decreased antioxidants (P<0.05); consequently, psoriasis-like cell models were well established. In the presence of PCs, nevertheless, the proliferation rate and number of psoriasis-like cells evidently decreased (P<0.01), accompanied with enhanced HO-1 and antioxidants, and lowered OS/inflammatory indicators as well as phosphorylated JAK2/STAT3/PI3/AKT (P<0.01). Similar changes appeared after LY294002 pretreatment, regardless of PCs or not. But after ZnPP pretreatment with or without PCs, the opposite occurred.

**Conclusion:**

The study reveals that PCs can suppress psoriasis-like cell proliferation and reduce inflammatory/OS damage through PI3K/AKT inhibition and HO-1 activation, thus promising a candidate for PCs in treating psoriasis.

## 1. Introduction

Psoriasis is an immune-mediated, chronic inflammatory disease, histologically characterized by the abnormal proliferation/differentiation of keratinocytes (KCs), excessive angiogenesis and inflammatory cells infiltration in the dermis [1,2]. Currently, 2%–5% of the worldwide population suffers from psoriasis that negatively affects both individual mental and physical health [3,4]. Although the precise etiology of psoriasis keeps obscure, factors including environment, genetics, immunology, inflammation and oxidative stress (OS) are mostly implicated; particularly, inflammation and OS both contribute to the development of psoriasis [5–7]. Experiments *in vitro* revealed that the levels of OS-related indicators in psoriatic lesions and serum were significantly aberrant, presenting as insufficient antioxidants [e.g. superoxide dismutase (SOD), glutathione peroxidase (GSH-Px), glutathione (GSH), catalase (CAT) and others] and excessive oxides [e.g. malondialdehyde (MDA), nitric oxide (NO), superoxide radical (O2-), etc] [8]. Furthermore, the expression of OS-related markers closely correlates with the severity and activity of psoriasis; patients with moderate to severe active psoriasis display a lower expression of SOD/GSH-PX and a higher expression of MDA than those with mild inactive psoriasis [9]. OS, particularly mild OS, not only encourages the activation and proliferation of several cells (KCs and T cells in special), but also initiates an aberrant inflammatory response [10]. Reactive oxygen species (ROS) generated from OS initially activate multiple inflammatory signaling pathways, such as janus kinase-signal transducer and activator of transcription (JAK/STAT), phosphoinositide 3-kinase/protein kinase B (PI3K/AKT), and so on [11]. Following that, dendritic cells (DCs) are stimulated and release various inflammatory cytokines, like interleukin (IL)-23 and tumor necrosis factor alpha (TNF-α), to facilitate the abnormal differentiation and activation of T lymphocytes that further induce the production of IL-17, IL-22, TNF-α and interferon-gamma (IFN-γ), and the activation of downstream signal STAT3 [12]; then, KCs are aroused to produce diverse cytokines [e.g. adenosine monophosphate (AMP) and Chemokine (C–C motif) ligand 20 (CCL20)] that mutually recruit neutrophils and reversely activate T lymphocytes, thereby forming an amplified inflammatory cascade [13]; alterations including KCs hyperproliferation/differentiation, angiogenesis and inflammatory cells infiltration would follow, eventually contributing to the occurrence of pathological changes and clinical symptoms of psoriasis [14]. Thus, inflammation and OS are key to psoriasis pathogenesis and would be as crucial targets for psoriasis treatment.

There are many therapeutic approaches to psoriasis available today, consisting of topical corticosteroids, ultraviolet (UV) light-based therapy, retinoid drugs, vitamin D analogs, immunosuppressants and biological agents; but most of them are frequently limited by their undesirable properties such as short-term efficacy, easy relapse, intolerable side effects, high-cost expenditure and so on [15]. As a result, an affordable, safe and effective treatment is critical to psoriasis healing. Natural plant extracts arrest the attention owing to their favorable bioactivities and few adverse effects. Proanthocyanidins (PCs), a typical representative of natural plant extracts derived from fruits, vegetables and seeds, stand out and exert diversified functions, covering anti-oxidation, anti-inflammation, anti-angiogenesis, anti-proliferation, immunological modulation and others [16,17]. Basing on their multiple activities and few adverse reactions, PCs have been widely applied in a variety of clinical fields [18,19]. Numerous studies have confirmed that PCs are good for controlling various disorders, such as autoimmune arthritis, diabetes, cardiovascular disease and squamous cell carcinoma, through fighting OS damage via inhibition of mitogen-activated protein kinase/nuclear factor kappa-B (MAPK/NF-κB) pathway and activation of heme oxygenase-1 (HO-1) signal [20–24]. Meanwhile, PCs availably curb the release of diverse inflammatory factors to halt the inflammatory response via blocking the JAK/STAT signal pathway [25]. What’s more, PCs not only restrain PI3K/AKT signal to increase the apoptosis and autophagy of cells, but also suppress vascular endothelial growth factor (VEGF) expression and endothelial cells migration to prevent the formation of new blood vessels [26]. Given the pathogenesis of psoriasis and the powerful functions of PCs, we suppose PCs would effectively work in psoriasis. To date, however, reports about PCs application in psoriasis rarely emerge. In our previous experiment, we found that PCs remarkably alleviated the clinical symptoms and pathological alterations of imiquimod (IMQ)-induced psoriasis-like mice, lowered the levels of ROS, MDA and inflammatory factors, and enhanced the expression of antioxidant enzymes, possibly involving the reduction of inflammatory response and OS damage [27]. Further to investigate the specific mechanism of PCs against psoriasis, the present study was carried out.

## 2. Materials and methods

### 2.1. Cell culture

HaCaT cells (ATCC, USA) grew in 1640 medium containing 10% fetal bovine serum and 1% penicillin/streptomycin (Gibco, USA) at 37°C/5% CO_2_ in a moist atmosphere, and were conventionally subcultured to 2–3 passages for further experiments.

### 2.2. Determination of the safe concentration of PCs

HaCaT cells were seeded into 6-well plates at a density of 1 × 10^6^ cells/well. After a 24-hour starvation culture, the original media was discarded. Then, five concentrations of PCs (Solarbio, Beijing) (30 μg/mL, 60 μg/mL, 90 μg/mL, 120 μg/mL, and 150 μg/mL) were added to the corresponding well plates. Forty-eight hours later, the CCK-8 kit was used to determine the safe PCs concentration for the following experiment.

### 2.3. Construction of the psoriasis-like cell models

HaCaT cells were inoculated at a density of 1 × 10^6^ cells/well in 6-well plates. The original media was discarded after a 24h-starvation culture. The cells, subsequently, were added with 3 mL/well of culture medium containing TNF-α (7.5 ng/mL; PROTEINTECH GROUP, USA) for another 48-hour culture to establish the psoriasis-like cell model. Finally, the supernate and cells were collected for detection.

### 2.4. Intervention of PCs to TNF-α induced psoriasis-like cell models

HaCaT cells were firstly seeded into 6-well plates at the density of 1 × 10^6^ cells/well and divided into five groups: normal group (no intervention), model group (TNF-α alone), low-concentration PCs group (TNF-α+50 μg/mL PCs), medium-concentration PCs group (TNF-α+75 μg/mL PCs) and high-concentration PCs group (TNF-α+ 100 μg/mL PCs). After a 24-hour starving culture, the original medium was abandoned. The latter three groups then received 3 mL/well of culture medium containing TNF-α (7.5 ng/mL) and different concentrations of PCs, whereas the normal group was given with 3 mL/well of culture medium alone and the model group with an equal-volume culture medium containing TNF-α (7.5 ng/mL). Subsequently, they all continued to be cultured for 48 h. Lastly, the supernate and cells were harvested for indicators analysis.

### 2.5. Application of inhibitors to the psoriasis-like cells

To clarify the specific mechanism of PCs on experimental psoriasis, psoriasis-like cells respectively underwent the pretreatment of LY294002 or ZnPP in a separate experiment. In this additional experiment, the psoriasis-like cell models were divided into three groups: TNF-α+PCs + LY294002/ZnPP group, TNF-α+PCs group and TNF-α+LY294002/ZnPP group. Briefly, the psoriasis-like cell models were pretreated with (or without) LY294002 (MCE, USA) for four hours before PCs intervention in 6-well plates; while the psoriasis-like cell models were pretreated with (or without) ZnPP (MCE, USA) for one hour prior to PCs intervention in 6-well plates.

### 2.6. CCK-8 assay for cell proliferation

The proliferation of cells was determined by the Cell Counting Kit-8 (CCK-8) assay (Solarbio, Beijing). The amount of formazan, produced from the reduction of CCK-8 by the living cells, was quantitated by monitoring absorbance at 450 nm. All the steps were performed following the instructions.

### 2.7. ELISA analysis for the inflammatory and OS-related biomarkers

Inflammatory biomarkers (including IL-17 and IL-23) and OS-related biomarkers (including CAT, SOD, GSH, MDA, and ROS) were measured in the supernatant of each group. ELISA assay kits for IL-17 and IL-23 and ROS were obtained from Beijing Andihuatai Bioengineering Institute, while those kits for SOD, CAT, GSH and MDA came from Nanjing Jiancheng Bioengineering Institute. All the parameters were determined under the manufacturers’ instructions.

### 2.8. Real-Time quantitative reverse transcription PCR (qRT-PCR) analysis

To measure the mRNA levels of *JAK2, STAT3, PI3K, AKT* and *HO-1*, total RNA was extracted from the cells of different groups by using the Trizol RNA extraction kit according to the manufacturer’s instructions (Ambion, USA). Target gene expressions were determined relative to those of *GAPDH* (internal control). The primers used were listed in Table 1. Besides, BLAST searches were performed and each accession number to gene primer was also exhibited in the table below.

**Table 1.** The primers of cellular protective genes.

Genes	Numbers	Sequence (5′-3′)		Length
*GAPDH*	NM_001256799	Forward	CATCATCCCTGCCTCTACTGG	259
		Reverse	GTGGGTGTCGCTGTTGAAGTC	
*JAK2*	NM_004972	Forward	TGCCAGAAACTTGAAACTTAAGTAT	218
		Reverse	CGCAATATAACTGTAAATCCTGTTC	
*STAT3*	NM_139276	Forward	GGCGTCACTTTCACTTGGGT	219
		Reverse	CTCTGGCCGACAATACTTTCC	
*PI3K*	NM_006218	Forward	GTCCTATTGTCGTGCATGTGG	292
		Reverse	TGGGTTCTCCCAATTCAACC	
*AKT*	NM_005163	Forward	TTCTATGGCGCTGAGATTGTGT	228
		Reverse	GCCGTAGTCATTGTCCTCCAG	
*HO-1*	NM_002133	Forward	GCCAGCAACAAAGTGCAAGA	100
		Reverse	TAAGGACCCATCGGAGAAGC	

### 2.9. Western blot analysis

For the detection of p-JAK2, JAK2, p-STAT3, STAT3, p-PI3K, PI3K, p-AKT, AKT and HO-1 protein levels, total proteins were separately extracted from the cells of each group by using the lysis and protein loading buffers. Pierce BCA Protein Assay was applied to determine the protein concentrations. The SDS-PAGE Gel system was established. The primary antibodies against phosphorylated JAK2 (p-JAK2, 1:1000 v/v, rabbit anti-human, anti-p-JAK2 monoclonal antibody, Abcam, UK), JAK2 (1:5000 v/v, rabbit anti-human, anti-JAK2 monoclonal antibody, Abcam, UK), p-STAT3 (1:2000 v/v, rabbit anti-human, anti-p-STAT3 monoclonal antibody, CST, USA), STAT3 (1:1000 v/v, rabbit anti-human, anti-STAT3 monoclonal antibody, Abcam, UK), p-PI3K (1:1000 v/v, rabbit anti-human, anti-p-PI3K polyclonal antibody, Bioworld, USA), PI3K (1:5000 v/v, mouse anti-human, anti-PI3K monoclonal antibody, Proteintech Group, USA), p-AKT (1:2000 v/v, rabbit anti-human, anti-p-AKT monoclonal antibody, CST, USA), AKT (1:2000 v/v, rabbit anti-human, anti-AKT polyclonal antibody, Proteintech Group, USA) and HO-1 (1:10000 v/v, rabbit anti-human, anti-HO-1 monoclonal antibody, Abcam, UK) were introduced. After incubation with respective secondary antibodies (1:10000 v/v, goat anti-rabbit IgG, Affinity Biosciences, Jiangsu; 1:10000 v/v, goat anti-mouse IgG, Affinity Biosciences, Jiangsu), signals were detected and analyzed by using a protein electrophoresis system (Bio-Rad Laboratories Inc., USA), automatic chemiluminescence analyzer (Tanon Science and Technology Ltd., China) and TANON GIS (Tanon Science and Technology Ltd., China). Anti-β-actin monoclonal antibody (mouse anti-human, Bioworld, USA) was used as an internal control.

### 2.10. Statistical analysis

Data were presented as mean ± SD and the statistical analysis was performed with SPSS 26.0. All data sets were tested for normalcy by Shapiro–Wilk test. Then analysis of variance (ANOVA) or Student's t-test was used to analyze between-group differences. Differences were regarded as statistical significance at *P* < 0.05. Graphpad Prism 8.0 software was employed for data graphing.

## 3. Results

### 3.1. The influence of PCs on normal cells

**Figure 1.**
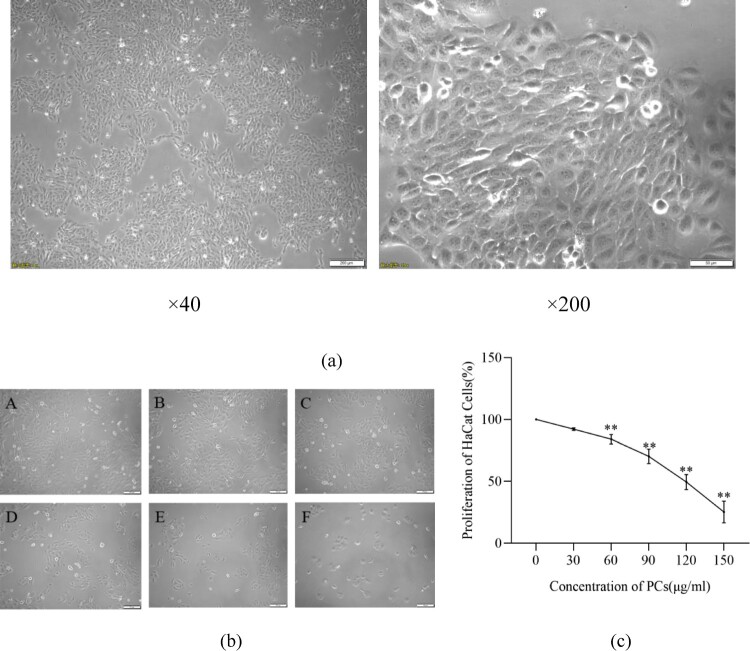
Influence of PCs on the morphology and proliferation of HaCaT cells. (a) Morphology of HaCaT cells; (b) Effect of PCs on the morphology of HaCaT cells; (c) Effect of PCs on the proliferation of HaCaT cells. A: 0 μg/mL; B: 30 μg/mL; C: 60 μg/mL; D: 90 μg/mL; E: 120 μg/mL; F: 150 μg/mL. Note: Compared with the control group, *P < 0.05, **P < 0.01.

The normal KCs (HaCaT cells) exhibited an adherent-growth characteristic and a paving stone-like morphology (Figure 1(a)). After 48-hour PCs intervention, no significant alterations, regardless of cell state or shape, emerged from the groups of 0 μg/mL, 30 μg/mL and 60 μg/mL, cells in which all adherently grew well (Figure 1(b)). In 90 μg/mL group, the majority of cells tightly attached to the wall and displayed a fine growth behavior; inversely, most cells in group of 120 μg/mL exfoliated in the medium, as well few adherent cells in group of 150 μg/mL (Figure 1(b)). Besides, the cell proliferative ratio decreased with the increase of PCs concentration (Figure 1(c)). The 50% inhibitory concentration (IC50) of PCs was determined to be 114.20 ± 12.82 μg/mL. As a result, the suitable concentrations of PCs at 50 μg/mL, 75 μg/mL and 100 μg/mL were chosen for future study.

### 3.2. Characteristics of TNF-ɑ-induced psoriasis-like cell model

**Figure 2.**
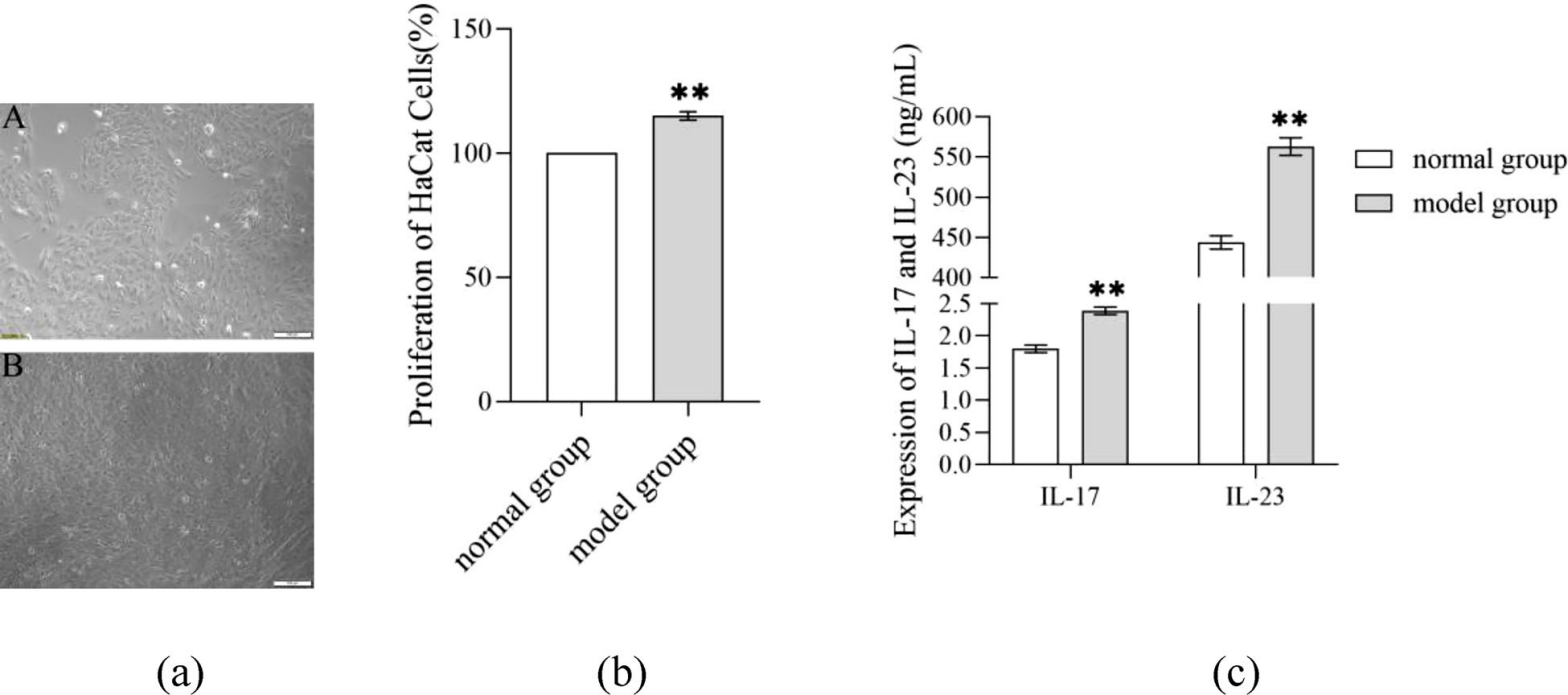
Features of TNF-ɑ-induced psoriasis-like cell model. (a) Morphology of HaCaT cells under the intervention of TNF-α; A: normal group, B: model group; (b) Effect of TNF-α on the proliferation of HaCaT cells; (c) Effect of TNF-α on inflammatory factors in the supernatant of psoriasis-like cells. Note: Compared with the normal group, **P < 0.01.

Following TNF-ɑ induction for 48 h, psoriasis-like cells greatly outnumbered normal cells. These psoriasis-like cells adherently grew as paving shape and arranged more tightly. Compared with the normal group, the cell proliferation ratio and levels of IL-17, IL-23, ROS and MDA in the model group remarkably enhanced (*P* < 0.05), while the expression of SOD, CAT and GSH lowered greatly (*P* < 0.01) (Figure 2) (Table 2).

**Table 2.** Levels of OS-related indicators in supernatant of psoriasis-like cell model (X ± S).

Group	ROS (ng/mL)	MDA (nmol/mL)	SOD (U/mL)	CAT (U/mL)	GSH (nmol/mL)
Normal Group	6.61 ± 0.39	8.15 ± 0.70	21.64 ± 1.18	1.51 ± 0.14	47.83 ± 2.02
Model Group	7.55 ± 0.66*	10.74 ± 0.41**	10.87 ± 2.04**	0.57 ± 0.13**	26.83 ± 1.62**

Note: Compared with the normal group, *P < 0.05, **P < 0.01.

### 3.3. PCs inhibited the hyperproliferation of TNF-ɑ-induced psoriasis-like cells

**Figure 3.**
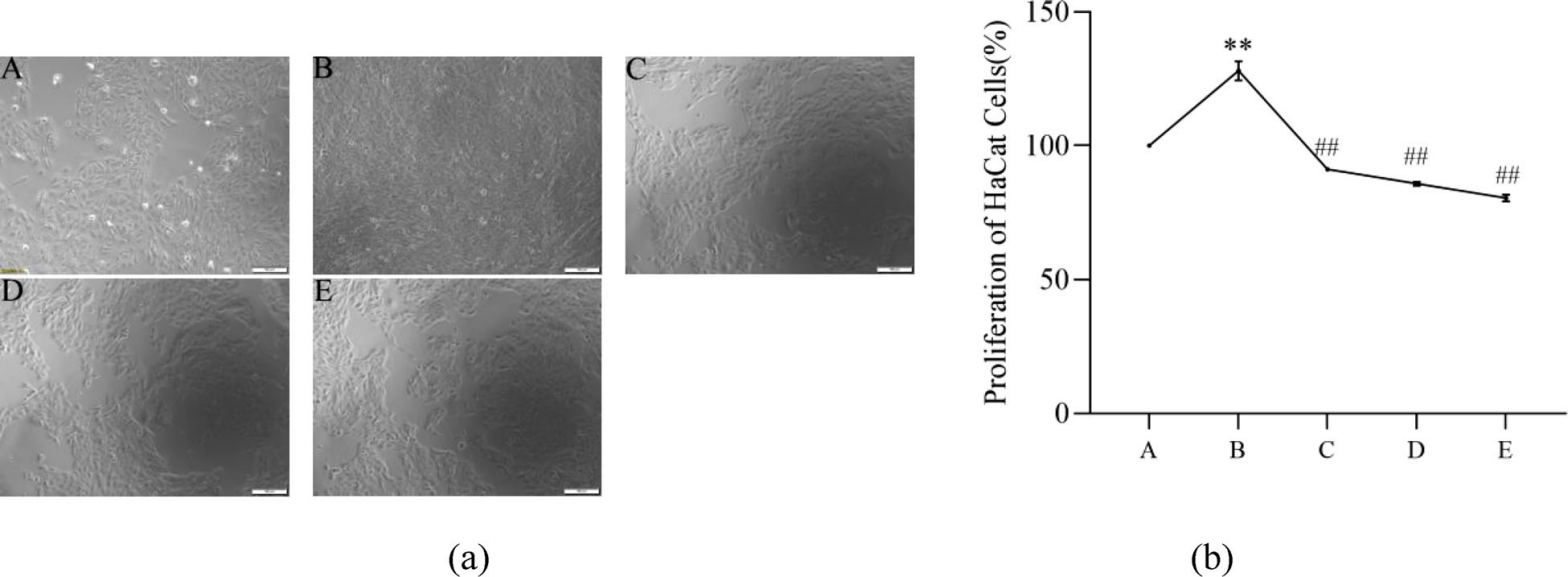
Effect of PCs on psoriasis-like cell models. (a) Effect of TNF-α and PCs on the morphology of HaCaT cells; (b) Effect of TNF-α and PCs on the proliferation of HaCaT cells. A: normal group, B: model group, C: low-concentration PCs group, D: medium-concentration PCs group, E: high-concentration PCs group. Note: Compared with the normal group, **P < 0.01; Compared with the model group, ##*P* < 0.01.

In comparison to the model group, the number and density of cells in the low-concentration PCs group went down, and the intercellular space slightly broadened; while the intercellular space in the medium-concentration PCs group further enlarged; in the high-concentration PCs group, however, the intercellular space obviously widened (Figure 3(a)). Although TNF-ɑ alone significantly enhanced the cell proliferative rate compared to the normal group (*P* < 0.01), PCs in a concentration-dependent tendency remarkably reduced TNF-ɑ-induced cell hyperproliferation (*P* < 0.01) (Figure 3(b)).

### 3.4. PCs attenuated the oxidative damage of TNF-ɑ-induced psoriasis-like cells

**Figure 4.**
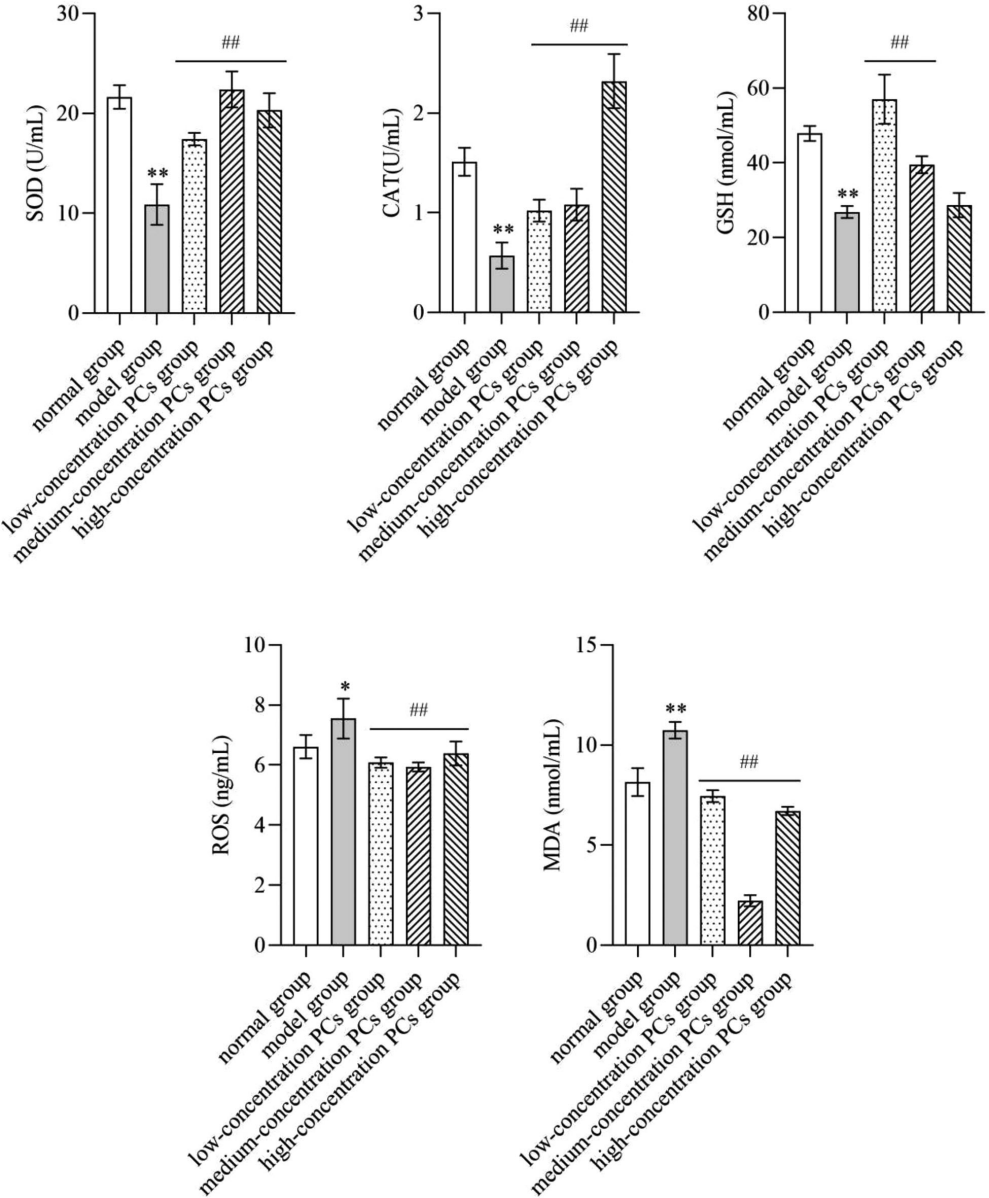
Levels of oxidative indicators in supernatant of different-concentration groups. Note: Compared with the normal group, *P < 0.05, **P < 0.01; Compared with the model group, ##*P* < 0.01.

TNF-ɑ remarkably lowered the levels of SOD, CAT and GSH, but elevated those of MDA and ROS (*P* < 0.05) (Figure 4). Nevertheless, PCs treatment reversed these conditions; namely, PCs in different degrees increased the activities of SOD, CAT and GSH, and decreased the expression of MDA and ROS (*P* < 0.01) (Figure 4).

### 3.5. PCs mitigated the inflammatory response of TNF-ɑ-induced psoriasis-like cells

**Figure 5.**
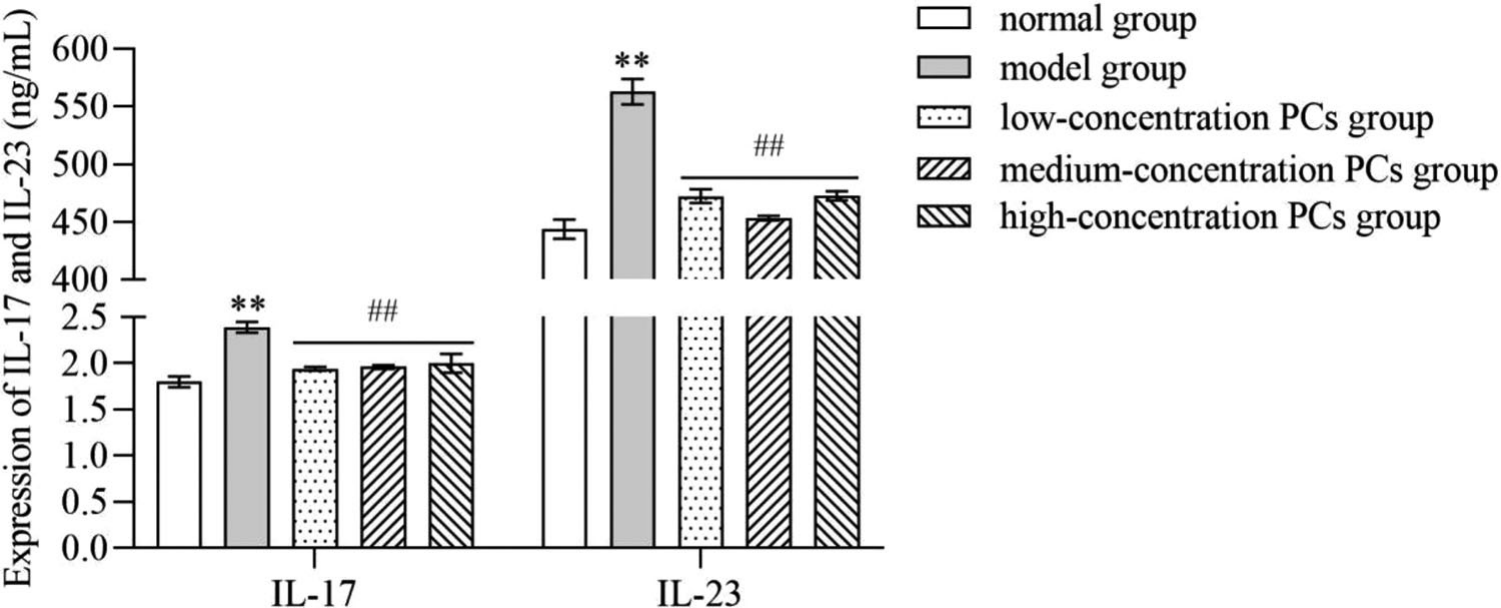
Levels of inflammatory factors in supernatant of different-concentration groups. Note: Compared with the normal group, **P < 0.01; Compared with the model group, ##*P* < 0.01.

TNF-ɑ significantly heightened the expression of inflammatory cytokines like IL-17 and IL-23 (*P* < 0.01) (Figure 5). PCs, however, markedly cut down the expression of these inflammatory indicators induced by TNF-ɑ (*P* < 0.01) (Figure 5); whereas, little significant difference existed in different-concentration PCs groups (*P* > 0.05).

### 3.6. PCs regulated the expression of oxidative/inflammatory genes and proteins

**Figure 6.**
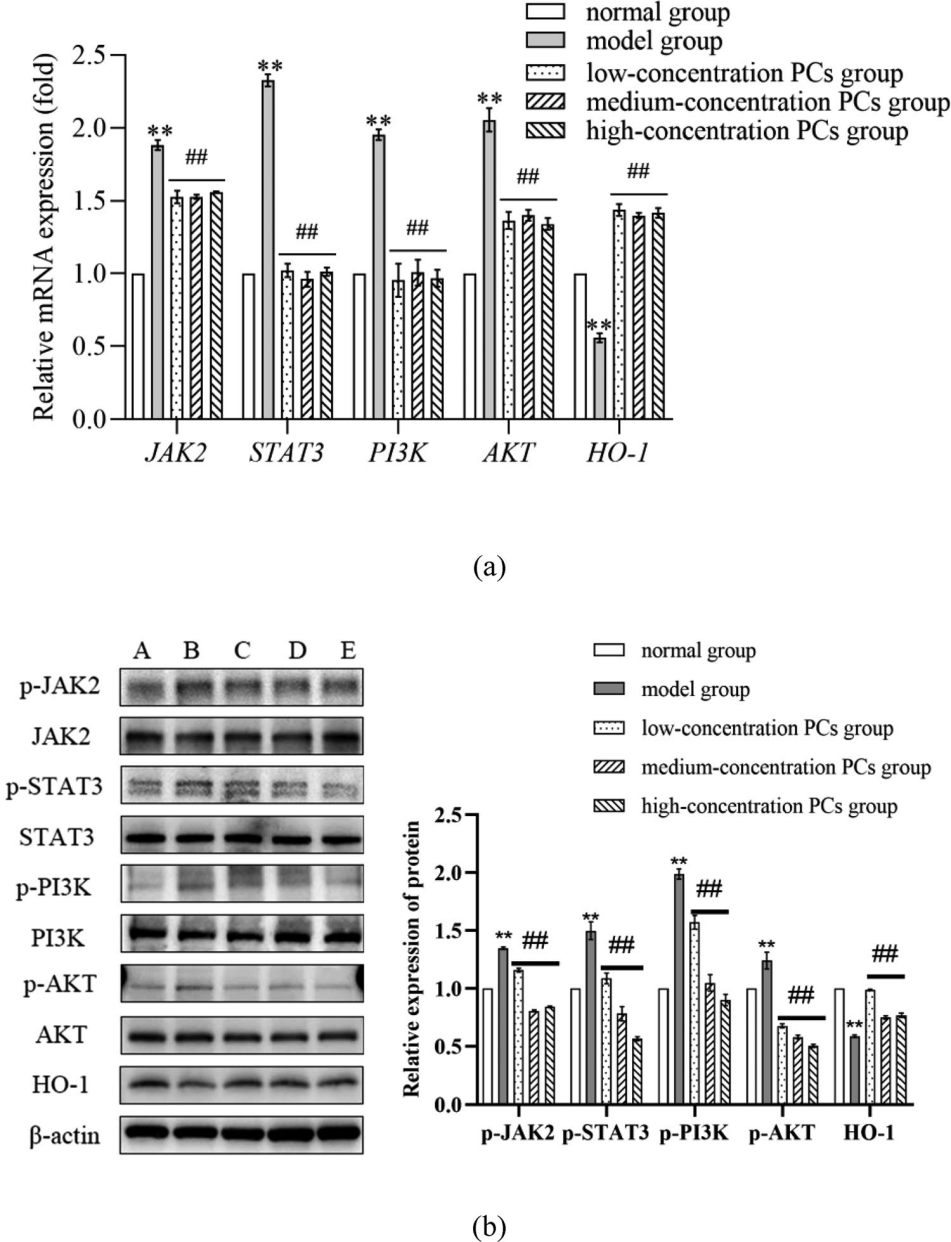
Expression of mRNA/protein different-concentration groups. (a) The mRNA relative expression of OS/inflammatory indicators in different groups; (b) The protein expression of OS/inflammatory indicators in different groups. A: normal group, B: model group, C: low-concentration PCs group, D: medium-concentration PCs group, E: high-concentration PCs group. Note: Compared with the normal group, **P < 0.01; Compared with the model group, #*P* < 0.05, ##*P* < 0.01.

After TNF-ɑ-induction, the mRNA and phosphorylated protein levels of JAK2, STAT3, PI3K and AKT obviously ascended, while those of HO-1 dramatically went down (*P* < 0.01) (Figure 6). Nevertheless, these phenomena were reversed by PCs treatment (*P* < 0.01) (Figure 6); PCs apparently lowered the mRNA and phosphorylated protein expressions of JAK2, STAT3, PI3K and AKT, but greatly enhanced those of HO-1 (*P* < 0.01) (Figure 6). Besides, the total protein expressions of JAK2, STAT3, PI3K and AKT exhibited little statistical significance among different groups (*P* > 0.05).

### 3.7. PCs inhibited PI3K/AKT pathway but stimulated HO-1 signal

**Figure 7.**
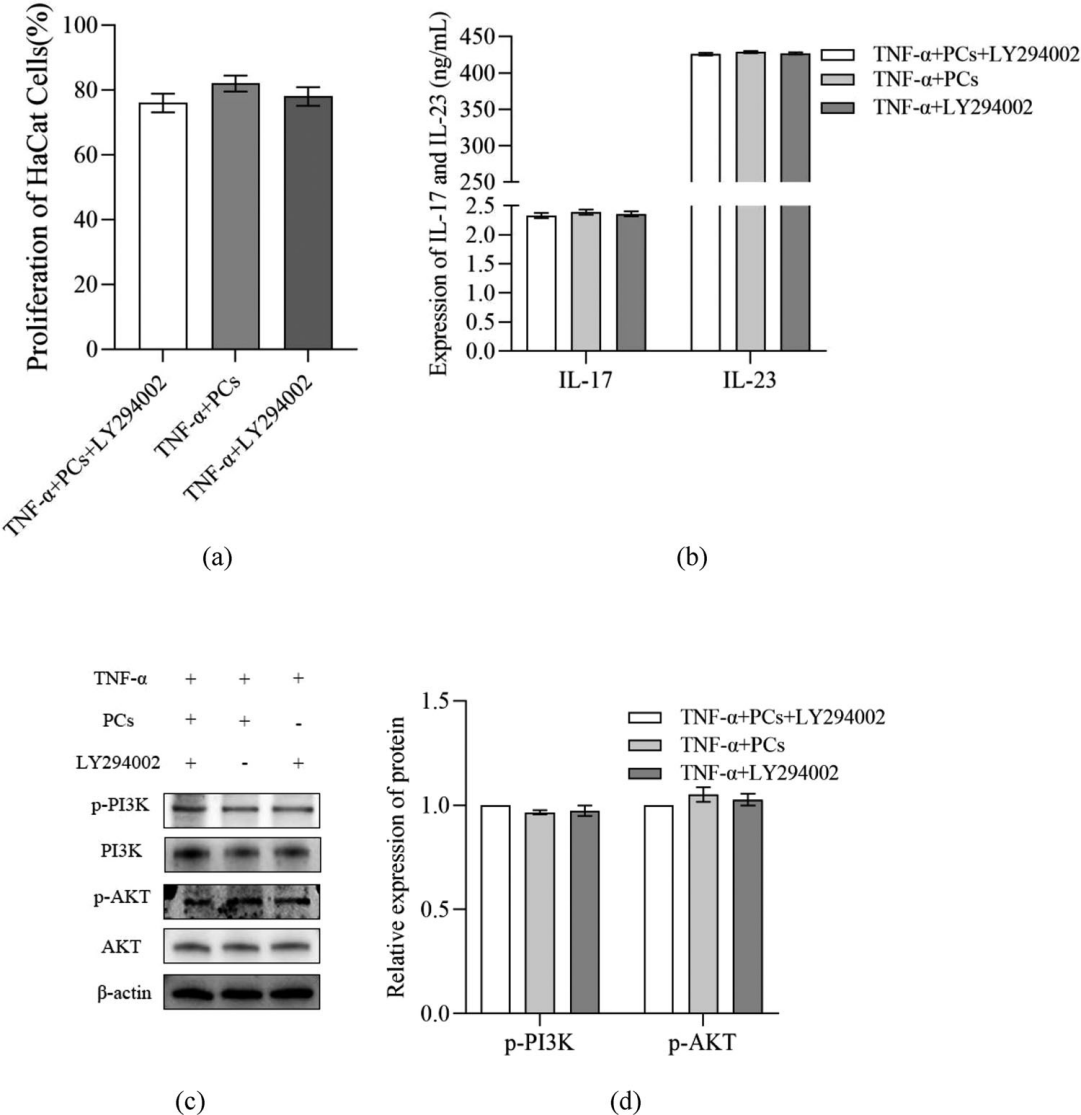
Effect of inhibitor of PI3K on cell proliferative activity and inflammatory factors expression in psoriasis-like cells treated with PCs. (a) Effect of PCs or/and LY294002 on the proliferation of cells; (b) Effect of PCs or/and LY294002 on the levels of inflammatory factors in different groups; (c) The protein band expression of p-PI3K and p-AKT in different groups; (d) The relatively quantitative expression of p-PI3K and p-AKT proteins in different groups. Note: Compared with the TNF-α+ PCs group, **P < 0.01.

**Figure 8.**
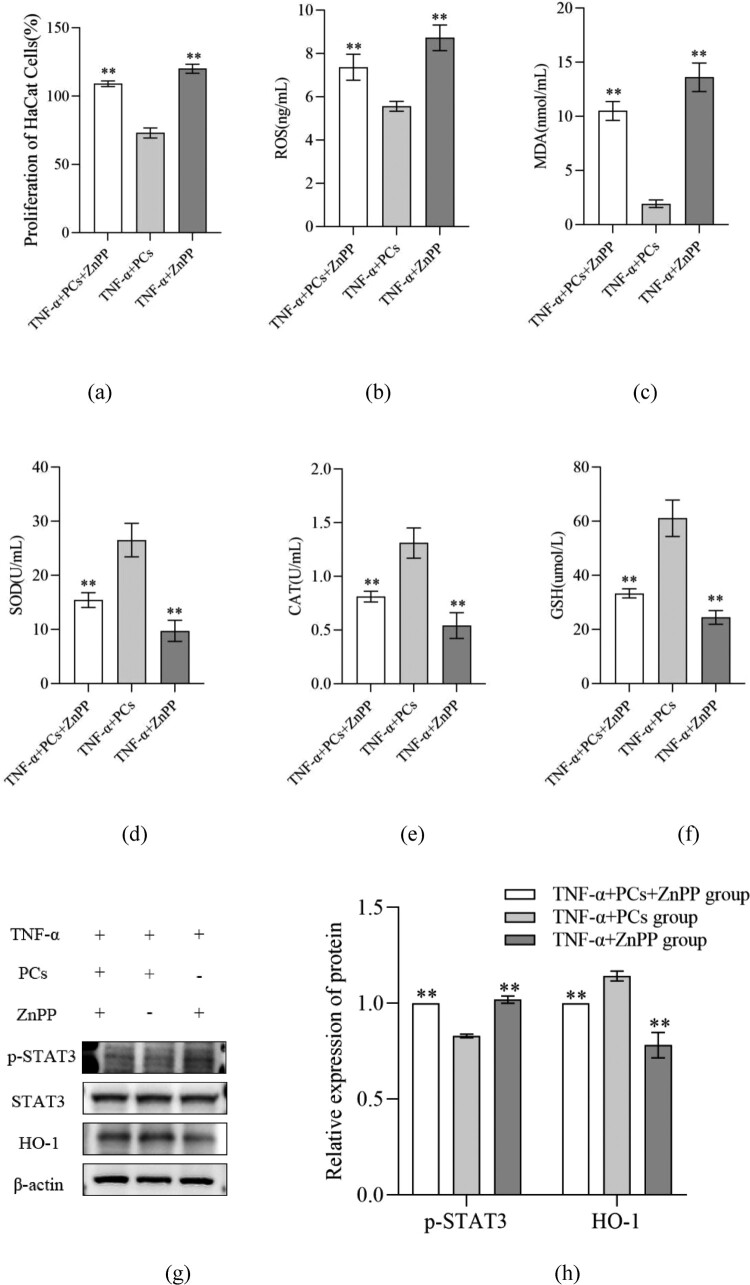
Effect of inhibitor of HO-1 on cell proliferative activity and OS factors expression in psoriasis-like cells treated with PCs. (a) Effect of PCs or/and ZnPP on the proliferation of cells; (b) Level of ROS in supernatant of different groups; (c) Level of MDA in supernatant of different groups; (d) Level of SOD in supernatant of different groups; (e) Level of CAT in supernatant of different groups; (f) Level of GSH in supernatant of different groups; (g) The protein band expression of HO-1 and p-STAT3 in different groups; (h) The relatively quantitative expression of HO-1 and p-STAT3 proteins in different groups. Note: Compared with the TNF-α+ PCs group, **P < 0.01.

To clarify the mechanisms of PCs against the psoriasis-like cells, the components of PI3K/AKT pathway and HO-1 signal were investigated. After treatment with PCs, cells in different-concentration groups displayed an obvious decrease in proliferative activity, inflammatory indicators (including IL-17, IL-23, p-PI3K, p-AKT and p-STAT3), and OS-related parameters (like ROS and MDA), along with an increase in SOD, CAT, GSH and HO-1. Pretreatment with PI3K inhibitor (LY294002) later, meanwhile, similar changes still emerged regardless of PCs intervention or not; namely, cell proliferation and levels of IL-17, IL-23, p-PI3K and p-AKT dropped in different degree; moreover, no significant difference existed between the PCs group and PI3K inhibitor group (Figure 7). In the presence of HO-1 inhibitor (ZnPP), yet, above alterations rarely occurred; conversely, cell proliferative activity and the levels of ROS, MDA and p-STAT3 increased, whereas those of SOD, CAT, GSH and HO-1 decreased (*P* < 0.01) (Figure 8). Notably, the expression of HO-1 protein fell off and that of p-STAT3 went up more obviously in the ZnPP group than those in the group of PCs plus ZnPP. Nevertheless, no significant difference was found in the total protein expression of STAT3 among different groups (*P* > 0.05) (Figure 8).

## 4. Discussion

In the present study, we successfully uncovered the mechanism underlying the effect of PCs against the psoriasis-like cell models. First, PCs were extremely effective in suppressing the hyperproliferation of TNF-ɑ-induced psoriasis-like cells. Next, PCs remarkably decreased the levels of oxidative indicators and inflammatory factors, containing MDA, ROS, IL-17, and IL-23, but increased the activities of antioxidative indexes like SOD, CAT, and GSH. Lastly, the psoriasis-like cell models, treated with PCs in the absence of LY294002 and ZnPP, showed low expressions of p-PI3K, p-AKT, and p-STAT3 proteins, along with a high expression of HO-1 protein. Moreover, above phenomena likewise occurred even in the presence of PI3K inhibitor. These conditions, however, were reversed in the presence of HO-1 inhibitor; that was, going with an increase in MDA, ROS, IL-17 and IL-23, and a decrease in SOD, CAT and GSH. These findings indicated that PCs had a strong resistance to TNF-ɑ-induced psoriasis-like cell models via blocking PI3K/AKT pathway and the exciting HO-1 signal.

PCs, belonging to plant flavonoids, exert various active functions, e.g. anti-oxidation, anti-inflammation, anti-angiogenesis, anti-proliferation, and immunomodulation [28]. Although PCs are safe and non-toxic,it still needs to ensure the safe concentration of PCs when investigating their therapeutic effects; while in this concentration range, PCs would scarcely cause unacceptable damage to normal cells. So to determine the safe scope, different-concentration PCs were initially used to intervene in the normal cells. Our results indicated that IC50 of PCs was 114.20 ± 12.82 μg/mL, which coincided with the previous findings [29]; according to the IC50 value, the concentrations of 50, 75, and 100 μg/mL were optimal and chosen for further experiments.

Because the hyper-proliferation and aberrant differentiation of KCs are the primary features of psoritic histopathology, they frequently serve as the ideal cells for constructing psoriasis-like cell models [30]. In the present study, therefore, we selected KCs (HaCaT cells) with TNF-α induction to build a psoriatic cell model. As one of the crucial inflammatory factors in psoriasis progression, TNF-α could promote KCs proliferation, aggravate inflammation and facilitate ROS production/accumulation through mediating inflammatory and OS-related signal pathways e.g. HO-1, JAK/STAT, PI3K/AKT, NF-κB, etc [31]. According to the data from documents, a 7.5 ng/mL concentration of TNF-α was adopted to establish psoriasis-like cell models in our experiment. The results revealed that alterations of cell models, including cellular morphology and proliferation as well as the level of inflammation and OS, squared with the previous outcomes, indicating the successful establishment of psoriasis-like cell models in our study [32].

Basing on the screener of safe-concentration drug and the construction of the cell model, we employed different concentrations of PCs to intervene in the psoriasis-like cell models. As a result, it showed that PCs could apparently suppress the proliferation of psoriasis-like cells in a concentration-dependent tendency, which was similar to the findings from Decean and García-Pérez et al. They found *in vitro* that PCs prevented the proliferation of KCs as well other cells and restored normal epithelial keratinization [29,33]. The above results uncovered that PCs had a certain effect on TNF-α-induced psoriasis-like cells and could stop the excessive proliferation of psoriatic cells, which were in agreement with our previous animal experimental findings [27], implying that PCs are quite effective in experimental psoriasis.

Nevertheless, it needs to clarify how PCs do work. Recently, increasing reports have shown that there is a decrease in the antioxidants containing SOD, CAT and GSH from psoriasis patients, whereas an increase in oxides like ROS, MDA and NO [34]; this imbalance of the oxidative/anti-oxidative system triggers OS and excites OS/ inflammation-related signals, which stimulate several cells, mainly T cells and DCs, to secrete a variety of inflammatory mediators/cytokines (e.g. IL-17, IL-23, VEGF, TNF-α, TGF-β, and IFN-γ), eventually leading to KCs hyper-proliferation, inflammatory cells infiltration, and neovascularization [35,36]. In our study, however, PCs could shift this unequilibrium; namely, PCs remarkably increased the levels of antioxidants, e.g. SOD, CAT, and GSH, and decreased the expression of oxides and inflammatory factors like IL-17, IL-23, ROS, and MDA in the psoriasis-like cells. Miao et al. likewise demonstrated the potent ability of PCs to lower MDA and elevate SOD and GSH, along with the prevention of OS-mediated damage and the reinforcement of antioxidant defence [37,38]. In addition, it has been proved that PCs effectively equilibrate T cells and attenuate inflammatory factors (such as IL-17, IL-21, IL-22, IL-23, etc.), suggesting that PC have a favorable prospect in the treatment of inflammatory and autoimmune diseases [39]; Park et al., for instance, discovered that PCs obviously alleviated the clinical symptoms of collagen-induced arthritis in mice via inhibition of IL-17 inflammatory mediator [40]. Our current outcomes were also consistent with the above findings, which confirmed the anti-oxidative and anti-inflammatory effects of PCs in psoriasis-like cell models. However, the specific mechanism underlying PCs against psoriasis kept unclear.

Related studies have verified that some crucial signals, especially PI3K, AKT, JAK, STAT and HO-1, are closely implicated in the pathogenesis of psoriasis [41–43]. PI3K/AKT is a critical signaling pathway for cell survival and proliferation, actively regulating cell growth, proliferation and metabolism [44]. Activation of PI3K/AKT promotes the occurrence and progression of psoriasis, while inhibition of them decays KCs hyper-proliferation and inflammatory factors expression in psoriasis [45]. Besides, the activation of JAK/STAT, STAT3 in particular, is deeply involved in psoriasis, accelerating psoriatic KCs proliferation, angiogenesis, and T cells abnormal differentiation [46,47]. More importantly, HO-1, as the crucial component of the cellular anti-oxidative system, goes together with the prognosis of psoriasis [43]. In their experiments, Ma et al. found that inhibition of HO-1 exacerbated the symptoms of psoriasis-like animals, suggesting that HO-1 might be protective to bodies against psoriasis [48]. Similar studies also confirmed that activated HO-1 could significantly mitigate IMQ-induced psoriasis-like inflammation by blocking STAT3 signaling pathway [49]. These demonstrate that the signaling pathways of JAK/STAT, PI3K/AKT and HO-1 are critical to psoriasis, particularly the latter two. To clarify the specific mechanism of PCs against psoriasis, therefore, we detected the levels of JAK/STAT, PI3K/AKT and HO-1. Our results showed that JAK/STAT and PI3K/AKT levels remarkably decreased while HO-1 expression greatly increased in different- concentration PCs groups compared with those in the model group, which were in line with the previously documented findings from Song and Zhang et al.; they verified that PCs could lower JAK/STAT and PI3K/AKT expression to inhibit the proliferation and migration of cells and eliminate ROS as well inflammation [50,51]; Sun et al. also found that HO-1 apparently elevated in the presence of PCs [37]. It, in the present study, revealed that PCs resistance to experimental psoriasis might be through suppressing JAK/STAT and PI3K/AKT pathways and activating HO-1 signal, especially PI3K and HO-1 signals. Further to confirm the role of PI3K and HO-1 in PCs treatment, we employed the appropriate inhibitors to block PI3K and HO-1 signals. The current results showed that, in the presence of a PI3K inhibitor, cell proliferation rate and expression of IL-17, IL-23 and p-PI3K/p-AKT reduced regardless of PCs treatment or not, but little significant difference existed when compared to those in PCs treatment alone. This indicates that inhibition of PI3K signaling pathway is partly responsible for PCs against psoriasis. On the other hand, PCs treatment facilitated a decrease in the cell proliferation rate and the expression of ROS, MDA and p-STAT3, as well as an increase in SOD, CAT, GSH and HO-1; however, the above phenomena failed to appear in the presence of HO-1 inhibitor regardless of PCs treatment or not; instead, the cell proliferation rate and the expression of ROS, MDA and p-STAT3 ascended, while the expression of SOD, CAT, GSH and HO-1 descended. Nevertheless, the expression of HO-1 protein was much lower in the group of HO-1 inhibitor alone than that in the group of PCs plus HO-1 inhibitor, whereas p-STAT3 expression exhibited an opposite trend to HO-1. Both implied the molecular mechanisms of PCs regulating HO-1 possibly involving multiple signaling pathways. It, alternatively, verifies that HO-1 signal activation is one of the major mechanisms of PCs against psoriasis in our experiment. Consequently, it demonstrated that PCs reduced STAT expression primarily via inhibition of PI3K/AKT and activation of HO-1, thereby alleviating the inflammatory response and OS damage, prohibiting psoriasis-like cell proliferation, and ultimately controlling psoriasis.

## 5. Conclusion

In conclusion, our findings reveal that PCs excellently work in inhibiting the over-proliferation of TNF-α-induced psoriasis-like cells and attenuating inflammatory and OS damage, the underlying mechanism of PCs against psoriasis mainly involving the inhibition of PI3K/AKT and the activation of HO-1. Hence, modulation of PI3K/AKT and HO-1 would benefit to improve the effect of PCs against psoriasis, implying a potential value of PCs in control of psoriasis. However, cell models *in vitro* cannot simulate the real condition of human psoriasis completely. Besides, it fails to uncover the effect of PCs on T cells through the present model. Thus, Th1/Th2 shift studies e.g. co-culture of T cells and HaCat in further experiments should be required to verify the role of PCs in treating human psoriasis.

## Data Availability

The data that support the findings of this study are available from the corresponding author upon reasonable request.

## Abbreviations

**Abbreviations**: KCs: keratinocytes; OS: oxidative stress; SOD: superoxide dismutase; GSH-Px: glutathione peroxidase; CAT: catalase; MDA: malondialdehyde; NO: nitric oxide; O^2−^: superoxide radical; ROS: reactive oxygen species; JAK/STAT: janus kinase-signal transducer and activator of transcription; PI3K/AKT: phosphoinositide 3-kinase/protein kinase B; DCs: dendritic cells; IL-23:, interleukin-23; TNF-α: tumor necrosis factor alpha; IFN-γ: interferon-gamma; AMP: adenosine monophosphate; CCL20: Chemokine (C–C motif) ligand 20; UV: ultraviolet; PCs: proanthocyanidins; MAPK/NF-κB: mitogen-activated protein kinase/nuclear factor kappa-B; HO-1: heme oxygenase-1; VEGF: vascular endothelial growth factor; IMQ: imiquimod; ANOVA: analysis of variance; TGF-β: transforming growth factor-β
